# Improved pulmonary function is associated with reduced inflammation after hybrid whole‐body exercise training in persons with spinal cord injury

**DOI:** 10.1113/EP090785

**Published:** 2023-01-09

**Authors:** Brandon A. Yates, Robert Brown, Glen Picard, J. Andrew Taylor

**Affiliations:** ^1^ Cardiovascular Research Laboratory Spaulding Rehabilitation Hospital Cambridge MA USA; ^2^ Department of Physical Medicine and Rehabilitation Harvard Medical School Boston MA USA; ^3^ Indiana Center for Musculoskeletal Health Indiana University School of Medicine Indianapolis IN USA; ^4^ Pulmonary and Critical Care Medicine Unit and Department of Medicine Massachusetts General Hospital Boston MA USA

**Keywords:** exercise physiology, paraplegia, tetraplegia

## Abstract

The current study was designed to test the hypotheses that (1) reducing systemic inflammation via a 12‐week functional electrical stimulation rowing exercise training (FESRT) prescription results in augmented pulmonary function, and (2) the magnitude of improvement in pulmonary function is inversely associated with the magnitude of systemic inflammation suppression in persons with sub‐acute (≤2 years) spinal cord injury (SCI). We conducted a retrospective analysis of a randomized controlled trial (NCT#02139436). Twenty‐one participants were enrolled (standard of care (SOC; *n* = 9) or FESRT (*n* = 12)). The exercise prescription was three sessions/week at 70–85% of peak heart rate. A two‐way analysis of covariance and regression analysis was used to assess group differences and associations between pulmonary function, log transformed high‐sensitivity C‐reactive protein (hsCRP_log_) and white blood cell count (WBC). Following FESRT, clinically significant improvements in forced expiratory volume in 1 s (FEV_1_; 0.25 (0.08–0.43) vs. −0.06 (−0.26 to 0.15) litres) and forced vital capacity (0.22 (0.04–0.39) vs. 0.08 (−0.29 to 0.12) litres) were noted and systemic WBC (−1.45 (−2.48 to −0.50) vs. 0.41 (−0.74 to 1.56) μl) levels were suppressed compared to SOC (mean change (95% confidence interval); *P* < 0.05). Additionally, both ΔhsCRP_log_ and ΔWBC were predictors of ΔFEV_1_ (*r* ^2^ = 0.89 and 0.43, respectively; *P* < 0.05). Twelve weeks of FESRT improves pulmonary function and reduces WBC in persons with sub‐acute SCI. The potency of FESRT to augment pulmonary function may depend on adequate suppression of systemic inflammation.

## Abstract (highlights)

- **What is the central question of this study?**
  Does 12 weeks of functional electrical stimulation (FES) rowing exercise training lead to suppressed systemic inflammation and an improvement in pulmonary function in persons with sub‐acute spinal cord injury (SCI)?
- **What is the main finding and its importance?**
  Twelve weeks of FES rowing exercise improves pulmonary function and the magnitude of improvement is associated with reductions in inflammatory biomarkers. Thus, interventions targeting inflammation may lead to better pulmonary outcomes for person with sub‐acute SCI.

## 1 INTRODUCTION

The National Spinal Cord Injury Model Systems surveillance data indicate that long‐term survival of persons with spinal cord injury (SCI) has not improved in the last 30 years (Shavelle et al., 2015). It is plausible that the high prevalence of respiratory disease associated with SCI accounts, in part, for the unchanged rates of mortality in this population (Bloom et al., 2020; Myers et al., 2007). Indeed, within 2 years post SCI, precipitating risk factors for respiratory pathology are present across multiple organ systems and go untreated (Bloom et al., 2020; Giangregorio & McCartney, 2006; Kern et al., 2008; Postma et al., 2013; Spungen et al., 2003): reduced lean muscle mass, lung compliance, and exercise capacity and increased chest wall stiffness, physical inactivity, and adipose accumulation. Collectively, these risk factors contribute to an unfavourable systemic pro‐inflammatory profile, which is negatively associated with respiratory health in persons with SCI (Hart et al., 2016, 2017). For instance, Hart et al. demonstrated that key pulmonary function parameters, forced expiratory volume in 1 s (FEV_1_) and forced vital capacity (FVC), were inversely related to systemic pro‐inflammatory biomarkers (Hart et al., 2016, 2017). Similarly, low physical activity is associated with both poorer lung function and expiratory capacity (Montesinos‐Magraner et al., 2016). Further, respiratory inefficiencies are a limiting factor of peak aerobic capacity (${\dot{V}}_{O_{2}\mathrm{peak}}$) in persons with SCI, especially those with higher level lesions (Battikha et al., 2014). Yet, it remains unclear if reducing systemic inflammation via increased physical activity or exercise training promotes an improvement in pulmonary health outcomes in persons with SCI.

Although aerobic exercise is recognized as the most potent non‐pharmacological rehabilitative treatment for persons with SCI (Myers et al., 2007), the effectiveness of an exercise prescription is dependent on training parameters such as duration, intensity, modality and frequency, which can be problematic for individuals with SCI. This is, in part, because of an inability to engage substantial muscle mass to achieve threshold intensity levels that promote training‐related adaptations compared to able‐bodied persons (Alves et al., 2021). To overcome this challenge, functional electrical stimulation (FES) exercise devices have been leveraged to improve exercise performance in persons with SCI (Vivodtzev & Taylor, 2021), but to our knowledge only one report has examined systemic inflammatory responses to FES exercise training. In that report Allison and colleagues demonstrated that 12 weeks of exercise training with FES cycling resulted in no improvement in the inflammatory profile of persons with SCI (Allison et al., 2016), but no data were presented demonstrating that the exercise regimen increased physical activity and/or exercise capacity. As such, it cannot be ascertained if the lack of an improvement in a participant's inflammatory profile was due to lack of an adequate exercise training stimulus. Indeed, exercise intensity is positively related to the abundance of anti‐inflammatory mediators in response to exercise training (Petersen & Pedersen, 2005) and it is well accepted that adequately dosed exercise prescriptions promote improvements in exercise capacity. Therefore, given the aforementioned relationships between activity level, pulmonary function, and inflammation, the present study was designed to test the hypotheses that (1) 12 weeks of a hybrid whole‐body FES modality, FES rowing exercise training (FESRT), reduces systemic pro‐inflammatory biomarkers and improves pulmonary function, and (2) improvements in pulmonary function are associated with reductions in systemic inflammation in persons with SCI.

## 2 METHODS

### 2.1 Ethical approval

All participants provided written informed consent and this study was approved by the Spaulding Rehabilitation Hospital Institutional Review Board (Protocol no. 2013P000604). The study conformed to the standards set by the *Declaration of Helsinki*, except for registration in a database.

### 2.2 Study design and population

We conducted a retrospective analysis of participants from a randomized controlled trial comparing 6 months of FESRT to standard of care (SOC) treatments in persons with sub‐acute SCI (≤2 years) (Afshari et al., 2022). From the parent randomized control trial participant pool, 22 individuals, who also underwent routine resting pulmonary function testing, at baseline and 3 months, were screened into this study. Although >1 year is typically considered chronic SCI, we operationally determined sub‐acute as ≤2 years post‐injury because the rate of skeletal muscle atrophy (Giangregorio & McCartney, 2006; Spungen et al., 2003) and pulmonary function decline (Postma et al., 2013) is greatest during this period and as such represents a critical period for therapeutic intervention. In the parent study, participants were randomized to the following: FESRT, arms‐only row training (no lower extremity FES (active control)), or no formal exercise (time control). Due to no statistical or clinically meaningful differences in pulmonary function or systemic inflammation (primary outcomes) between the active control (*n* = 6) and time control (*n* = 4) at 3 months, we combined the groups and operationally defined them as SOC. All participants had a physical examination and were cleared to participate by an SCI board certified physiatrist. Study exclusion criteria were: <18 or >40 years of age, history of smoking, cardiovascular, pulmonary or renal disease, currently prescribed cardioactive medications, clinically diagnosed with diabetes, cancer, epilepsy or neurological disorders (other than SCI), currently suffering from grade 2 or greater pressure sore(s), lower extremity contractures, peripheral nerve compressions, or rotator cuff tears that restricted or limited the ability to perform the rowing exercise. Study staff who analysed the primary outcomes variables were blinded to a participant's group allocation.

### 2.3 Exercise regimen

The exercise program was identical to what we have previously reported (Afshari et al., 2022). Briefly, all participants allocated to arms‐only exercise or FESRT were prescribed exercise at a dosage of three 30‐min sessions a week with the goal of reaching an exercise intensity of 70–85% of peak heart rate. On average, both control and treatment group's adherence to the programme was approximately 68% (25/36 sessions attended). FESRT participants’ baseline relative ${\dot{V}}_{O_{2}\mathrm{peak}}$ values averaged 5.2 METS (metabolic equivalent of task; moderate intensity) whereas for those in the SOC, who performed arms‐only exercise, it was 3.7 METS (low intensity). Therefore, during the intervention FESRT participants were inherently able to train at a higher daily exercise absolute intensity compared to SOC.

### 2.4 Peak cardiopulmonary exercise testing

Following familiarization of either arms‐only or FES rowing exercise, graded exercise tests were performed at baseline and repeated at about 3 months. A certified clinical exercise physiologist designed individualized work progressions based on the participants’ fitness level and motor function capabilities. After an individualized warm‐up, participants performed rowing exercise in stages until reaching volitional fatigue at approximately 8–12 min. Incremental increases in work output were employed every 1–2 min and lower limb electrical stimulation was adjusted accordingly to ensure full knee extension throughout the entire test. American Heart Association clinical acceptability criteria guidelines and procedures for cardiopulmonary exercise testing were utilized for the collection and validation of all gas‐exchange variables (Balady et al., 2010).

### 2.5 Spirometry

All testing was performed utilizing a 10‐litre spirometer (Collins, Braintree, MA, USA) and in accordance with modified American Thoracic Society and European Respiratory Society standards for acceptable and reproducible efforts in SCI (Kelley et al., 2003) prior to verifying resting maximal pulmonary function efforts.

### 2.6 Pro‐inflammatory biomarker collection and processing

Participants arrived at the laboratory fasted (>8 h) prior to all blood sample collections. Blood was drawn from the antecubital vein into an EDTA tube. Blood samples were externally analysed for high sensitivity C‐reactive protein (hsCRP) and white blood cell count (WBC) at a certified commercial laboratory (Quest Diagnostics, Marlborough, MA, USA).

### 2.7 Statistical analysis

Baseline observations >3 standard deviations (SD) were re‐evaluated on the basis of instrument precision and clinical implications to determine outliers. The Shapiro–Wilks test was used to assess normality of data. Homogeneity of variance was assessed by Levene's test on all continuous variables at baseline. Between‐group changes in ${\dot{V}}_{O_{2}\mathrm{peak}}$, pulmonary function and pro‐inflammatory biomarkers were assessed by a two‐way (group × time) repeated measures analysis of variance (ANOVA) or analysis of co‐variance (ANCOVA) with a Bonferroni *post hoc* correction for multiple comparisons. Partial eta square (η_p_^2^) effect sizes were calculated for interactive and main effects and Cohen's *d* effect size estimates and 95% confidence intervals (CI) were derived for within‐group differences. Pearson's correlation coefficient was used to assess the relationship between significant (*P* < 0.05) changes in ${\dot{V}}_{O_{2}\mathrm{peak}}$, pulmonary function and pro‐inflammatory outcomes. Based on significant correlations between the primary outcomes, a linear regression analysis was performed to further explore the associations among key outcomes. All data are reported as adjusted mean difference (95% CI) unless stated otherwise and SPSS Statistics version 27 (IBM Corp., Armonk, NY, USA) was used for statistical analyses.

### 2.8 Sample size calculation

Since a change of ≤8% or ≤150 ml in FEV_1_ or FVC is within the normal variability of spirometry (Kelley et al., 2003; Pellegrino et al., 2005), we determined a priori that a change in FEV_1_ or FVC of ≥12% or ≥200 ml would be considered a clinically meaningful change in respiratory function (Pellegrino et al., 2005). G*Power software was used to calculate sample size needed to detect an effect size (*f* = 0.5) of exercise on lung function (Montesinos‐Magraner et al., 2016; Neefkes‐Zonneveld et al., 2015) and systemic inflammation (Neefkes‐Zonneveld et al., 2015) for an ANOVA: repeated measures, within–between interaction analysis. At an a priori two‐sided α level of <0.05, 0.90 power and a correlation of 0.8 among repeated measures, the sample size required for each group was eight participants.

## 3 RESULTS

### 3.1 Participants

Clinical demographics at baseline are summarized in Table 1. Time since injury differed between groups at baseline (*P* = 0.01). Since time since injury is a determinant of lung volumes in this population (Stepp et al., 2008), it was treated as a co‐variate in general linear model statistical testing for ${\dot{V}}_{O_{2}\mathrm{peak}}$, spirometry and pro‐inflammatory outcome measures. Further, one participant was removed from the SOC group due to a baseline hsCRP value lying 3.64 standard deviations above the mean and the clinical implications of the reported value (44.2 mg/l; normal: ≤3 mg/l). Therefore, 21 participants (*n* = 12 (FESRT) vs. *n* = 9 (SOC)) were included in this retrospective analysis.

**TABLE 1:** Clinical characteristics of study participants.

	FES (*n =* 12)	SOC (*n* = 9)
Age (years)	29 (6)	29 (6)
Weight (kg)	76.0 (13.0)	81.9 (12.0)
Height (cm)	173.0 (9.4)	179.8 (6.8)
Body mass index (kg/m^2^)	25.4 (4.3)	25.3 (3.3)
Female (*n* (%))	2 (18.2)	1 (10)
Time since injury (years)	0.9 (0.5)	0.7 (0.2)
AIS grade (*n* (%))		
A	7 (58)	5 (56)
B	5 (42)	3 (33)
C	—	1 (11)
Level of injury (*n* (%))		
Tetraplegia	7 (58)	4 (44)
Paraplegia	5 (42)	5 (56)
Pulmonary function (% predicted)		
Forced vital capacity	62 (25)	69 (24)
Slow vital capacity	59 (22)	63 (25)
Inspiratory capacity	69 (23)	73 (29)
Expiratory reserve volume	40 (29)	44 (29)
Maximal voluntary ventilation	86 (40)	83 (49)
Forced expiratory volume in 1 s	62 (23)	67 (23)
Peak inspiratory flow	57 (32)	59 (39)
Peak expiratory flow	57 (18)	54 (17)
Pro‐inflammatory biomarkers		
High sensitivity C‐reactive protein (mg/l)	3.5 [6.8]	2.0 [3.8]
White blood cell count (μl)	8.5 (2.2)	6.5 (2.3)

*Note*: All values are either mean (SD) or median [interquartile range] unless stated otherwise.

### 3.2 Primary outcome

#### 3.2.1 Pulmonary function

**FIGURE 1:**
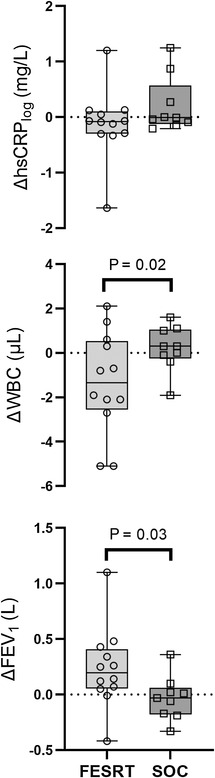
Within‐group mean differences in log transformed high sensitivity C‐ reactive protein, white blood cell count and forced expiratory volume in 1 s. Box plots illustrate the median and interquartile range. Whiskers describe the minimum and maximum recorded values. *P*‐values represent significant group × time interactions between FESRT (*n* = 12) and SOC (*n* = 9) groups. Significance was determined via repeated measures ANCOVA with Bonferroni corrections. FESRT, functional electrical stimulation row exercise training; FEV_1_, forced expiratory volume in 1 s; hsCRP_log_, log transformed high‐sensitivity C‐reactive protein; SOC, standard of care; WBC, white blood cell count.

Absolute changes (pre vs. post) in pulmonary function variables are shown in Table 2. Interaction (group × time) effects were observed for absolute FVC (*F*_1,18_ = 5.36; *P* = 0.03; η_p_^2^ = 0.23) and FEV_1_ (*F*_1,18_ = 5.64; *P* = 0.03; η_p_^2^ = 0.24; Figure 1). FESRT participants’ FVC (220 ml (0.04–0.39); *P* = 0.02) and FEV_1_ (250 (0.08–0.43) ml; *P* = 0.01) improved, whereas the SOC participants did not change (Table 2). All resting pulmonary function values are presented in Table 2.

**TABLE 2:** Between‐ and within‐group changes in pulmonary function and peak aerobic capacity parameters.

Measure		FES	ES (95% CI)	SOC	ES (95% CI)	Interaction *P*‐value
Peak aerobic capacity outcomes						
${\dot{V}}_{O_{2}}$ (l/min)	Pre	1.4 (0.4)	0.79 (0.12, 1.42)	1.0 (0.3)	0.46 ( −0.25, 1.13)	0.14
	Post	**1.6 (0.6)** ^†^		1.1 (0.3)		
${\dot{V}}_{O_{2}}$ (ml/kg/min)	Pre	18.1 (4.8)	0.69 (0.05, 1.31)	12.8 (4.6)	0.29 (−0.38, 0.95)	0.19
	Post	20.1 (6.2)		13.3 (4.6)		
${\dot{V}}_{E}$ (l/min)	Pre	45.7 (14.3)	0.66 (0.02, 1.28)	44.3 (14.3)	0.24 (−0.43, 0.90)	0.8
	Post	50.3 (13.9)		48.5 (17.4)		
O_2_ pulse (ml/kg/bpm)	Pre	8.8 (2.1)	1.18 (0.37, 1.83)	7.5 (1.4)	0.67 (−0.08, 1.38)	0.08
	Post	**10.2 (2.6)** ^†^		8.1 (1.6)		
HR (bpm)	Pre	160 (19)	0.07 (−0.50, 0.64)	139 (34)	−0.10 (−0.76, 0.56)	0.81
	Post	159 (25)		140 (33)		
Resting pulmonary function outcomes						
FVC (l)	Pre	2.91 (1.21)	0.72 (0.71, 1.35)	3.33 (0.95)	−0.19 (−0.85, 0.47)	**0.03** ^*^
	Post	3.11 (1.14)		3.26 (0.99)		
FEV1 (l)	Pre	2.41 (0.91)	0.65 (0.01, 1.26)	2.75 (0.81)	−0.16 (−0.82,0.50)	**0.03** ^*^
	Post	2.64 (0.91)		2.71 (0.81)		
SVC (l)	Pre	2.74 (1.03)	0.43 (−0.17, 1.02)	3.09 (1.10)	−0.10 (−0.75, 0.56)	0.27
	Post	2.89 (1.24)		3.04 (1.00)		
IC (l)	Pre	2.11 (0.74)	0.08 (−0.49, 0.64)	2.32 (0.69)	−0.5 (−0.70, 0.61)	0.64
	Post	2.14 (0.85)		2.30 (0.65)		
ERV (l)	Pre	0.62 (0.43)	0.54 (−0.08, 1.14)	0.75 (0.53)	−0.05 (−0.70, 0.61)	0.25
	Post	0.80 (0.52)		0.74 (0.48)		
MVV (l)	Pre	85.5 (40.4)	0.21 (−0.37, 0.78)	83.3 (49.0)	−0.15 (−0.81, 0.51)	0.56
	Post	89.1 (40.6)		80.0 (46.5)		
PIF (l/min)	Pre	3.45 (1.81)	−0.08 (−0.65, 0.49)	3.57 (1.99)	−0.31 (−0.97, 0.37)	0.75
	Post	3.32 (2.05)		3.36 (1.99)		
PEF (l/min)	Pre	4.95 (1.61)	0.45 (−0.16, 1.03)	4.98 (1.34)	0.60 (−0.13, 1.30)	0.97
	Post	5.28 (1.54)		5.44 (1.53)		

All values are adjusted mean (SD) unless stated otherwise. Bolded values represent statistical significance (*P* < 0.05). ^*^Statistically significant interaction effects. ^†^Significant within‐group time effect (*P* < 0.05). Significance was determined by repeated measures ANCOVA with Bonferroni corrections. Abbreviations: CI, confidence interval; ERV, expiratory reserve volume; ES, Cohen's *d* effect size estimate; HR, heart rate; IC, inspiratory capacity; MVV, maximum voluntary ventilation; PEF, peak expiratory flow; PIF, peak inspiratory flow; SVC, slow vital capacity; ${\dot{V}}_{E}$, minute ventilation.

#### 3.2.2 Pro‐inflammatory biomarkers

Interaction (group × time) effects were observed for WBC (*F*_1,18_ = 6.77; *P* = 0.02; η_p_^2^ = 0.27). FESRT participants experienced an almost 4‐fold greater reduction (*P* = 0.01) in WBC (−1.49 (−2.48 to −0.50) μl; Cohen's *d* = −0.58 (95% CI: −1.12 to 0.04)) compared to the SOC group (0.41 (−0.74 to 1.56) μl; Cohen's *d* = 0.21 (95% CI: −0.46 to 0.86); Figure 1). Due to failing the assumption of normality, hsCRP values were log_10_ transformed (hsCRP_log_) prior to statistical analyses. No time (*F*_1,18_ = 1.13; *P* = 0.30; η_p_^2^ = 0.06) or interaction (*F*_1,18_ = 2.36; *P* = 0.14; η_p_^2^ = 0.12) effects were noted for hsCRP_log_ (Figure 1).

#### 3.2.3 Correlation and regression analysis

**FIGURE 2:**
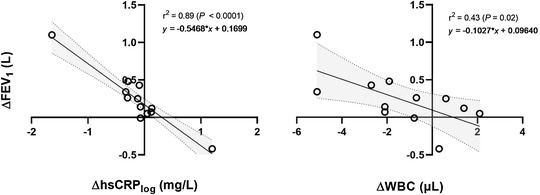
Linear regression analysis of changes in high sensitivity C‐reactive protein, white blood cell count and forced expiratory volume in 1 s following FESRT (*n* = 12). Shaded area represents the 95% confidence intervals. FEV_1_, forced expiratory volume in 1 s; hsCRP_log_, high sensitivity C‐reactive protein; WBC, white blood cell count.

A bivariate correlation analysis was performed comparing the change in primary pulmonary outcomes with changes in cardiorespiratory and inflammatory biomarkers that achieved statistical significance or had biological plausibility based on extant literature (Hart et al., 2017). The outcome of this analysis was the following: ΔWBC (μl) was significantly related to ΔFEV1 (l) (*r* = −0.65, *P* = 0.02) and ΔhsCRP_log_ (mg/l) (*r* = 0.61; *P* = 0.04); ΔhsCRP_log_ (mg/l) was significantly related to ΔFVC (l) (*r* = −0.66; *P* = 0.02) and ΔFEV1 (l) (*r* = −0.95; *P* < 0.0001); and ΔFEV_1_ (l) was significantly related to ΔFVC (l) (*r* = 0.78; *P* = 0.003). Based on the strength of the relationship between ΔFEV1 and both ΔWBC (μl) and ΔhsCRP_log_ (mg/l), a linear regression analysis was performed to examine if ΔhsCRP_log_ or ΔWBC was a significant predictor of ΔFEV_1_. The linear regression revealed that both ΔhsCRP_log_ and ΔWBC were significant predictors (*P* < 0.0001 and *P* = 0.02, respectively); but ΔhsCRP_log_ explained almost double the amount of variance in ΔFEV_1_ (*r* ^2^ = 0.89) compared to ΔWBC (*r* ^2^ = 0.43; Figure 2).

### 3.3 Secondary outcomes

#### 3.3.1 Peak aerobic capacity

Changes in ${\dot{V}}_{O_{2}\mathrm{peak}}$ outcomes are shown in Table 2. A main effect of time was observed for absolute (*F*_1,18_ = 4.86; *P* = 0.04; η_p_^2^ *=* 0.21) but not relative (*F*_1,18_ = 1.88; *P* = 0.19; η_p_^2^ *=* 0.10) ${\dot{V}}_{O_{2}\mathrm{peak}}$. Examination of simple main effects revealed that FESRT increased ${\dot{V}}_{O_{2}\mathrm{peak}}$ (0.21 (0.08–0.34) l/min; *P* = 0.004) but SOC was unchanged (0.06 (−0.09 to 0.21) l/min; *P* = 0.44). Additionally, a main effect of time was shown for oxygen pulse (O_2_ pulse – surrogate for stroke volume; *F*_1,18_ = 4.71; *P* = 0.04; η_p_^2^ = 0.21). Examination of simple effects revealed that FESRT participants significantly increased O_2_ pulse (1.51 (0.79–1.13) ml/kg/bpm; *P* < 0.0001) and SOC did not (0.51 (−0.29 to 1.37) ml/kg/bpm; *P* = 0.19).

## 4 DISCUSSION

We sought to examine the influence of 12 weeks of FESRT on systemic inflammation and pulmonary function in persons with sub‐acute SCI. Our novel findings are that 12 weeks of FESRT clinically improved FEV_1_ and FVC, but only the magnitude of augmented FEV_1_ was independently and inversely associated with ΔhsCRP_log_ and ΔWBC. Further, although both ΔhsCRP_log_ and ΔWBC were strong predictors of ΔFEV_1_, the former accounted for more than double the variance in ΔFEV_1_ compared to ΔWBC (89% vs. 43%, respectively). Taken together these findings indicate that, with respect to SOC, FESRT affords persons with SCI a unique advantage to improve pulmonary function potentially by reducing systemic inflammation. Notably, the results of the present study corroborate a recent report showing that 30 min of aerobic exercise three days/week is associated with improved respiratory function in 358 persons with SCI (Hoevenaars et al., 2022). However, the plausible mechanisms underpinning these findings likely reflect the inherent integrative physiological interactions between musculoskeletal, cardiovascular and respiratory systems that accompany regular whole‐body exercise training and as such warrant further discussion.

Lower extremity FES directs a greater fraction of cardiac reserve to the active musculature and reduces venous pooling in the lower extremity, via generation of a ‘muscle pump’, which results in an enhanced venous return, ventricular end‐diastolic volume, stroke volume and favourable cardiac morphological adaptations (Gibbons et al., 2016; Vivodtzev & Taylor, 2021). The increased O_2_ demand of active skeletal muscle stimulates an akin ventilatory response to mitigate ventilation–perfusion mismatching while performing FES‐rowing (Qiu et al., 2016; Vivodtzev & Taylor, 2021). However, persons with high‐level SCI exhibit limited ability actively increase ${\dot{V}}_{O_{2}\mathrm{peak}}$, because of an inefficient ventilatory response (Qiu et al., 2016), but co‐therapeutics – non‐invasive ventilation (Vivodtzev et al., 2020) or serotonin agonist (Vivodtzev et al., 2021) – may aid in overcoming this challenge. Further, performing FES‐rowing places competing demands on the thoracic complex to stabilize the spine and participate in dynamic torso flexion (Mahler et al., 1991) and elicits activation of the musculature involved in shoulder abduction (De Troyer & Boriek, 2011). In this regard, De Troyer et al. demonstrated that inadequate shoulder abduction reduced expiratory reserve volume by 60% and that strengthening of these muscles improved coughing (De Troyer & Boriek, 2011). Therefore, considering the changes in upper‐extremity force generation and strength (Terson de Paleville & Lorenz, 2015; Kim et al., 2014) alongside our observed improvements in FEV_1_ and FVC after FESRT, it is conceivable that neuromuscular function of the accessory muscles of respiration also improved during our study.

Collectively, the aforementioned mechanisms encourage higher‐intensity exercise training, which has been shown to enhance the anti‐inflammatory and immunomodulatory metabolic responses to acute and chronic aerobic exercise training (Chen et al., 2003). This is clinically relevant because the coalescing of accelerated denervated skeletal muscle proteolysis, inefficient respiratory airway mucus clearance, and increased physical inactivity and adipose accumulation consequent to SCI (Spungen et al., 2003) cultivates a local pro‐inflammatory milieu that may ‘spill over’ into the systemic circulation (Bloom et al., 2020) triggering various pathologies. For instance, under the aforementioned conditions, chronically elevated cytokine interleukin‐6 proceeds through *trans*‐signalling pathways upregulating hepatic CRP production, WBC and mucus hypersecretion within respiratory airways (Cerqueira et al., 2020). Conversely, after an acute exercise bout (in an intensity‐dependent fashion) systemic interleukin‐6 concentrations transiently increase markedly above basal levels but proceed through classic signalling pathways, inducing anti‐inflammatory and immunomodulatory cascades, resulting in increased skeletal muscle mass, neuromuscular function and aerobic capacity while reducing the systemic inflammatory burden. Taken together, our results indirectly suggest a greater anti‐inflammatory cytokine response after FESRT, compared to SOC, was elicited, which possibly mediates the association between both CRP and WBC with FEV_1_. However, the veracity of this hypothesis will need to be explored in future research.

### 4.1 Study limitations

Despite these compelling results our study has several limitations. First, because this was a retrospective analysis of a randomized control trial, we were not able to robustly assess changes in various pro‐inflammatory biomarkers consequent to the intervention. Notably, leptin, pro‐inflammatory adipose‐derived hormone, has also been demonstrated to also be inversely correlated with FEV_1_ but neither leptin nor fat mass or lean mass was assessed during this investigation. Nonetheless, a prior 12‐week FESRT study showed that, following training, leptin decreased in face of an 8% increase in relative ${\dot{V}}_{O_{2}\mathrm{peak}}$, but that study was underpowered to detect significant change in body composition (Jeon et al., 2010). Therefore, seeing that we observed an 11% increase in relative ${\dot{V}}_{O_{2}\mathrm{peak}}$, it is plausible that leptin decreased in parallel to the pro‐inflammatory biomarkers measured in our study. Further, physical activity level, dietary intake, and use of ibuprofen was not controlled for in this study. Thus, our results may be subject to confounding due to differences in activity levels outside of training sessions (Montesinos‐Magraner et al., 2016), daily caloric intakes or composition of diet, and/or non‐steroidal anti‐inflammatory drugs (Park et al., 2020). As such, caution must be taken in interpretation of our results.

### 4.2 Conclusions

In summary, our novel observations were the following: (1) clinically significant improvements in pulmonary function and suppression of pro‐inflammatory biomarkers are both achievable with 12 weeks of FESRT; (2) ΔFEV_1_ is inversely related to both ΔWBC and ΔhsCRP_log_; and (3) ΔhsCRP_log_ and ΔWBC are significant predictors of ΔFEV_1_ in persons with SCI. These findings signal the need for future investigations to delineate the musculoskeletal‐cardiovascular–respiratory inter‐relationships and to identify novel strategies that will enhance the potency of exercise prescriptions and potentially mitigate respiratory disease risk factors in persons with SCI.

## AUTHOR CONTRIBUTIONS

Brandon A. Yates, Glen Picard, Robert Brown and J. Andrew Taylor contributed to the conception of the work, the study design, interpretation of data, drafting of the work or revising it critically for important intellectual content. Brandon A. Yates and Glen Picard contributed to data acquisition and analysis of the work. All authors have read and approved the final version of this manuscript and agree to be accountable for all aspects of the work in ensuring that questions related to the accuracy or integrity of any part of the work are appropriately investigated and resolved. All persons designated as authors qualify for authorship, and all those who qualify for authorship are listed.

## CONFLICT OF INTEREST

None declared.

## Supporting information

Statistical Summary Document

## Data Availability

The data supporting the conclusions of this article will be made available by the authors upon reasonable request.
